# A novel DDAH-1 inhibitor improved sepsis-induced impairment in vasoreactivity to noradrenaline in a rat endotoxaemia model

**DOI:** 10.1186/cc10398

**Published:** 2011-10-27

**Authors:** Z Wang, V Taylor, R Stidwill, J Leiper, M Singer

**Affiliations:** 1 Bloomsbury Institute of Intensive Care Medicine, Department of Medicine, University College London, UK

## Introduction

In septic shock, iNOS activation and nitric oxide (NO) overproduction contribute to vascular hyporeactivity to adrenergic vasopressors. The consequent hypotension often necessitates high doses of catecholamine administration. However, this may lead to detrimental effects on tissue perfusion, immune function and myocardial function. Asymmetric dimethlyarginine (ADMA), an endogenous inhibitor of NO synthase, is extensively metabolised by dimethylarginine dimethylaminohydrolase (DDAH). Competitive inhibition of the DDAH-1 isoform should thus reverse hypotension but, as this isoform is absent in immune cells, it should not compromise the immune effects of NO. Hence, we investigated whether L257, a novel DDAH-1 inhibitor, could spare norepinephrine dosing in a rat endotoxic shock model.

## Methods

Anaesthetised, spontaneously breathing male Wistar rats (body weight 270 to 330 g) had their left carotid artery and right internal jugular vein cannulated for arterial pressure monitoring and fluid infusion, respectively. Then 40 mg/kg *Klebsiella pneumoniae *lipopolysaccharide was administered intravenously over 30 minutes followed by fluid resuscitation at a rate of 10 ml/kg/hour thereafter. When the mean arterial pressure fell over 20% below baseline, they received norepinephrine titrated to maintain arterial pressure at ±10% baseline. Thirty minutes post commencement of norepinephrine, animals were randomized to receive either L-257 (3 mg/kg bolus then infusion of 125 μg/hour) or, in controls, an equivalent volume of saline. Experiments were terminated 3 hours post commencement of norepinephrine titration, before which echocardiography was performed and serum samples were collected for biochemistry.

## Results

L-257-treated animals (*n *= 8) required a significantly lower total dose of noradrenaline over 3 hours compared with the eight control animals (38 ± 9 vs. 48 ± 4 μg, *P *< 0.05). The heart rate was significantly lower in the treatment group (*P *< 0.05), which associated with a trend of increased stroke volume and cardiac output. Serum BUN and urea were also significantly lower in the treatment group (*P *< 0.05, Table 1).

**Table 1:** 

Variable	NE	NE + L-257	*P *value
SV (ml/minute)	0.18 ± 0.04	0.23 ± 0.04	0.07
HR (beats/minute)	500 ± 15	449 ± 37	0.03
CO (ml)	90 ± 18	102 ± 19	0.31
U (mM)	16.6 ± 1.2	12.8 ± 1.7	0.01
BUN (mg/dl)	44.8 ± 3.7	35.9 ± 4.8	0.03
Cr (μM)	51.3 ± 15.2	31.8 ± 4.3	0.08
ALT (IU/l)	98 ± 79.8	71.3 ± 48.3	0.6

## Conclusion

In this acute endotoxic rat model, we demonstrate that DDAH-1 inhibition by L-257 could reduce norepinephrine dosage and ameliorate its harmful effects. This agent warrants further study as a putative therapy for septic shock.

